# 

**Published:** 1838-04-01

**Authors:** 


### CONTENTS

OF THE

### MEDICO-CHIRURGICAL REVIEW,

### No. LVI. APRIL 1, 1838,

### [BEING No. XVI. OF A DECENNIAL SERIES.]

### REVIEWS.

I.

DISEASES OF THE RECTUM.

I. A Treatise on the Malformations, Injuries, and Diseases of the Rectum and Anus. By GEORGE BUSHE, M.D.

II. On the Diseases of the Rectum. By JAMES SYME, F.R.S.E. .. 297

1. Hæmorrhoidal Affections .. 297
   - A. Congestion of the Hæmorrhoidal Vessels .. 297
   - B. Hæmorrhage .. 298
   - C. Internal Tumors .. 301
   - D. Tumors at the Verge of the Anus .. 307
   - E. External Hæmorrhoids .. 309
   - F. Causes of Hæmorrhoidal Tumors .. 309
   - G. Treatment of Hæmorrhoidal Affections .. 312
2. Prolapsus of the Rectum .. 323

II.

ON BUBO.

I. Practical Observations on the Venereal Disease, and on the Use of Mercury. By ABRAHAM COLLES, M.D.

II. A Treatise on the Venereal Disease and its Varieties. By WILLIAM WALLACE, M.R.I.A.

III. The Works of John Hunter, F.R.S., with Notes. Edited by JAMES F. PALMER, Esq. Vol. II. .. 329

1. Circumstances under which Bubo occurs .. 329
2. Treatment of Bubo .. 341
   - A. Treatment of Bubo in the Stage of Suppuration .. 347
3. Herpetic, or obstinate Ulcerations from Bubo .. 355
4. Sinus between the Scrotum and Thigh .. 359
5. Indolent Syphilitic Bubo .. 360

III.

PUBLICATIONS ON ANATOMY.

I. Observations on the Structure and Functions of the Spinal Cord. By R. A. GRAINGER, Esq.

II. Observations on the Ganglionic Enlargement of the Pneumogastric Nerve: the probable Function of that Ganglion, &c. By Mr. EDWARD COCK.

III. On the Distribution and probable Function of the superior and recurrent Laryngeal Nerves. By Mr. JOHN HILTON.

IV. Description of the Sacculus or Pouch in the Human Larynx. By Mr. JOHN HILTON .. 361

V. Cyclopædia of Anatomy and Physiology. Part 13 .. 378

1. Anatomy of the Spinal Cord .. 361
   - A. Theory of the Functions of the Sympathetic Nerve .. 365
   - B. Theory of Muscular Action .. 368
2. On the Ganglionic Enlargement of the Pneumogastric Nerve .. 372
3. On the Distribution and probable Function of the superior and recurrent Laryngeal Nerves .. 375

4. Description of the Sacculus or Pouch in the Human Larynx .. .. .. 376
5. Physiology of Generation .. .. .. .. .. .. .. .. .. .. .. .. .. 379

IV.

Notes on the Medical History and Statistics of the British Legion in Spain. By RUTHERFORD ALCOCK, K.T.S., &c. .. .. .. .. .. .. .. .. .. .. .. 387

V.

DISEASES OF THE WINDPIPE.

I. Observations on the Surgical Pathology of the Larynx and Trachea, including Remarks on Croup, Cynanche Laryngea, &c. By Mr. W. H. PORTER.. ..
II. A Treatise on the Diseases and Injuries of the Larynx and Trachea. By Mr. F. RYLAND .. .. .. .. .. .. .. .. .. .. .. .. .. ..
III. Traité Pratique de la Phthisie Laryngée. Par MM. TROUSSEAU et BELLOC .. } 393
1. Calcareous Degeneration of the Cartilages of the Larynx .. .. .. .. 394
2. Accidents from swallowing strong Acids, &c. .. .. .. .. .. .. .. .. 399
3. Foreign Bodies in the Larynx and Trachea .. .. .. .. .. .. .. .. 402
4. Wounds of the Larynx and Trachea.. .. .. .. .. .. .. .. .. .. .. 406
5. Bronchotomy as applicable to the Treatment of Asphyxia .. .. .. .. 410

VI.

COMPARATIVE ANATOMY.

NERVOUS SYSTEM OF THE AMPHIBIA.

I. Illustrations of the Comparative Anatomy of the Nervous System. By JOSEPH SWAN. Part III... .. .. .. .. .. .. .. .. .. .. .. .. ..
II. Outlines of Comparative Anatomy. By ROBERT E. GRANT, M.D. Part III. .. } 412
1. Comparative Anatomy of the Digestive Organs, Chyliferous and Sanguiferous Systems .. .. .. .. .. .. .. .. .. .. .. .. .. .. .. .. 417
A. Digestive Organs of the Cyclo-neurose, or Radiated Classes .. .. 418
B. Digestive Organs of the Articulata .. .. .. .. .. .. .. .. 421
C. Modifications of the Digestive Organs in the Higher Animals.. .. 424

VII.

Practical Observations on Nervous and Sympathetic Palpitation of the Heart, particularly as distinguished from Palpitation, the Result of Organic Disease, &c. &c. By JOHN CALTHROP WILLIAMS, M.D. .. .. .. .. .. .. .. .. .. .. .. 429

VIII.

PATHOLOGICAL ANATOMY.

I. Illustrations of the Elementary Forms of Disease. By ROBERT CARSWELL, M.D.
II. Lectures on the Morbid Anatomy of the Serous and Mucous Membranes. By THOMAS HODGKIN, M.D... .. .. .. .. .. .. .. .. .. .. .. .. .. } 440
1. Inflammation .. .. .. .. .. .. .. .. .. .. .. .. .. .. .. .. 441
A. Nature of Inflammation.. .. .. .. .. .. .. .. .. .. .. .. 441
B. Physical Characters of Inflammation .. .. .. .. .. .. .. .. 444
2. Morbid Anatomy of the Serous Membranes.. .. .. .. .. .. .. .. 450
A. Lesions of the Arachnoid .. .. .. .. .. .. .. .. .. .. .. 451
B. Lesions of the Pericardium .. .. .. .. .. .. .. .. .. .. .. 455

### PERISCOPE.

### Bibliographical Notices.

1. Traité Theorique et Pratique de la Derivation, &c.—A Theoretical and Practical Treatise on Derivation, &c. By L. F. Gondret, M.D. .. .. .. .. .. .. .. 457
2. Arsenicated Candles, Extracts from the Report of the Westminster Medical Society 459

3. Appendix to the Eighth Edition of the Pharmacologia, with Remarks. By J. A. Paris, M.D. .. 464

4. A Practical Treatise on the Curative Effects of Simple and Medicated Vapour applied locally, &c. &c. By James Wilson, M.D. .. 467

5. Elements of Anatomy. By Jones Quain, M.D. Parts I. and II. .. 469

6. Elements of Physiology. By J. Müller, M.D. Translated by William Baly, M.D. 469

7. Medicine and Surgery one inductive Science. By George Macilwain, Esq. .. 470

8. The Philosophy of the Eye. By Mr. John Walker .. 471

9. A Treatise on Ruptures. By William Lawrence, F.R.S... .. 471

10. Elements of Chemistry, including the recent Discoveries and Doctrines of the Science. By the late Edward Turner, M.D. .. 471

11. Practical Surgery. By Robert Liston, Surgeon .. 472

12. Institutes of Surgery. By Sir Charles Bell, K.G.H.. .. 472

### Clinical Review, and Hospital Reports.

KENT AND CANTERBURY HOSPITAL.

1. Fever from Prolonged Lactation .. 473

2. Gastro-Enteric Fever .. 475

3. Remittent Fever of Children .. 481

4. Bilious Remittent Fever .. 490

5. Intermittent Fever .. 496

WESTERN LYING-IN-HOSPITAL AND DISPENSARY—DUBLIN.

6. Medical Report, by Fleetwood Churchill, M.D. .. 506
A. Version Cases .. 510
B. Forceps Case .. 511
C. Crotchet Case .. 511

CITY OF LIMERICK INFIRMARY.

7. Injuries of the Knee-joint .. 512
1. Compound Dislocation of the Knee-joint .. 512
2. Incision into the Knee-joint—recovery .. 513
3. Wound of the Knee-joint—recovery.. .. 514
4. Wound of the Knee-joint—death .. 514
5. Wound of the Knee-joint—fatal Hæmorrhage from the Popliteal Artery . 514

ST. BARTHOLOMEW'S HOSPITAL.

8. Clinical Remarks on the best mode of Treating Encysted Tumors of the Eye-lids, containing Hair. By Mr. Lawrence .. 515
9. Treatment of Strumous Ophthalmia .. 516

GUY'S HOSPITAL.

10. Dr. Addison on Electricity in certain Convulsive and Spasmodic Diseases .. 517
11. Dr. Ashwell on the Speculum in Diseases of the Uterus .. 519

### Spirit of the Foreign Periodicals, &c.

1. M. Magendie on certain Auscultatory Phenomena .. 521
2. M. Magendie's Objections to the present Doctrines on the subject of Contagion .. 522
3. M. Magendie on some of the Phenomena of Absorption .. 527
4. Experiments on the Spermatic Animalculæ, and on some of the Causes of Sterility in Women .. 529
A. Importance of the Use of the Microscope in Physiological Enquiries .. 529
5. Case where the Fœtus was heard to cry before Birth .. 531
6. Remarks on Insanity—Important Distinction between two of its Forms .. 531
7. Increase of Insanity, with the Progress of Civilization .. 533
8. On the Duration of Life in France since the commencement of the present Century, by M. Bienaymé .. 534
9. Report on the Evils of the Factory System in France .. 535
10. Suggestions for the Improvement of the Morals and Manners of the Inhabitants of Large Towns in Germany .. 537
11. Rheumatism, Nature and proximate cause of—Employment of Opium in its Treatment, &c. .. 538
12. Cases Illustrative of Albuminaria, or Bright's Disease of the Kidneys .. 542

13. Pathology of Puerperal Fever (Metro-peritonitis). By Dr. Nonat.. .. .. .. .. 545

A. Lesions of the Uterus .. .. .. .. .. .. .. .. .. .. .. .. 546

B. Lesions of the Peritoneum .. .. .. .. .. .. .. .. .. .. .. .. 546

C. Lesions of the Digestive Tubes .. .. .. .. .. .. .. .. .. .. .. 547

14. On Phlebitis after Surgical Operations .. .. .. .. .. .. .. .. .. .. .. 547

15. On a peculiar Affection of the Fore-arm—Inflammation of the Sheaths, &c., of certain Muscles. By M. Maingault .. .. .. .. .. .. .. .. .. .. .. .. 549

16. On the Utility of very large Blisters in White-swellings and other Tumors of the Joints .. .. .. .. .. .. .. .. .. .. .. .. .. .. .. .. .. 550

17. Cases of Fracture.. .. .. .. .. .. .. .. .. .. .. .. .. .. .. .. 551

A. Fracture of the Patella by Muscular effort .. .. .. .. .. .. .. 551

B. Fracture of the Femur; spontaneous .. .. .. .. .. .. .. .. .. 551

18. Poisoning by Nitric Acid—subsequent Induration of the Pylorus and Death .. 551

19. Remarks on Epidemic Diseases .. .. .. .. .. .. .. .. .. .. .. .. .. 552

20. Cachexia Aquosa in Egypt .. .. .. .. .. .. .. .. .. .. .. .. .. .. 553

21. Raw Potatoes in Scurvy .. .. .. .. .. .. .. .. .. .. .. .. .. 553

22. Intense Cephalalgia, induced by the presence of Grubs in the Frontal Sinuses .. 553

23. Dysmenorrhea relieved by the Fumes of Carbonic Acid .. .. .. .. .. .. .. 554

24. Utility of the Fumes of Camphor in Rheumatism .. .. .. .. .. .. .. .. .. 554

25. Spontaneous Hydrophobia.. .. .. .. .. .. .. .. .. .. .. .. .. .. .. 555

26. Veratria—Remarks on the Employment of .. .. .. .. .. .. .. .. .. .. 555

27. Surgical Aphorisms. By Professor Walther . .. .. .. .. .. .. . .. 556

28. General Remarks on Ophthalmia .. .. .. .. .. . .. .. .. .. .. 558

29. Case of Xerophthalmia, with remarks on this rare disease. By M. Velpeau .. .. 557

30. On a new Means of Diagnosis between Amaurosis and Cataract. By M. Sanson .. 559

31. On the Treatment of Amaurosis by Cauterization of the Cornea .. .. .. .. .. 560

32. Extirpation of the Uterus .. .. .. .. .. .. .. .. .. .. .. .. .. .. 561

33. Radical Cure of Prolapsus Uteri .. .. .. .. .. .. .. .. .. .. .. .. .. 562

34. Treatment of Burns, by Mr. Velpeau, with diachylon plaster .. .. .. .. .. 562

35. Injections of Iodine in Hydrocele.. .. .. .. .. .. .. .. .. .. .. .. .. 563

36. Treatment of Strictures of the Urethra by Bougies coated with Calcined Alum .. 566

37. Spontaneous cure of an Aneurysm of the Iliac Artery .. .. .. .. .. .. .. 567

38. Perforation of the Vertebral Column by an Aneurysm of the Aorta, not suspected during life .. .. .. .. .. .. .. .. .. .. .. .. .. .. .. .. 568

### Spirit of the British and American Periodicals.

1. Some Observations on Abdominal Pulsation. By James Johnson, M.D. .. .. 569

2. Stricture of Œsophagus, communicating with the Trachea .. .. .. .. .. .. 571

5. Obscure Disease resembling the " Grand Climacteric." By Dr. Johnson .. .. 572

4. Hepatic Abscess bursting into the Colon and into the Lungs, with recovery. By Dr. Colledge .. .. .. .. .. .. .. .. .. .. .. .. .. .. .. .. .. 573

5. Phlebitis. By Dr. Monat .. . .. .. .. .. .. .. .. .. .. .. .. .. .. 574

6. Tympanitis Abdominalis .. .. .. .. .. .. .. .. .. .. .. .. .. .. 576

7. Dicksonianism at Home and Abroad .. .. .. .. .. .. .. .. .. .. .. .. 57[illegible]

8. Case of Hidrosis, or Hidrotic Fever. By Mr. J. C. Lever .. .. .. .. .. .. 57[illegible]

### Miscellanies.

1. Leeds Petition to Parliament .. .. .. .. .. .. .. .. .. .. .. .. .. 580

2. The Late Mr. Twining of Bengal.. .. .. .. .. .. .. .. .. .. .. .. .. 581

BIBLIOGRAPHICAL RECORD .. .. .. .. .. .. .. .. .. .. .. .. .. 582

### Appendix.

1. Mr. Guthrie's Clinical Lectures. 585

1. On Compound and Gun-shot Fractures of the Arm .. .. .. .. .. .. 602

2. On Compound and Gun-shot Fractures of the Thigh .. .. .. .. .. .. 608

3. On the Excision of the Head of the Thigh-bone .. .. .. .. .. .. .. 613

4. On Excision of the Elbow-joint.. .. .. .. .. .. .. .. .. .. .. .. 617

2. Breach of Etiquette in taking Fees from Medical Men .. .. .. .. .. .. .. 620

3. Negative Virtues; or, a new Method of rising on the Ruin of our Neighbours .. 622

4. Bye-laws, relating to Education, &c.—Royal College of Surgeons in Ireland .. 624

5. Liquor Lyttæ .. .. .. .. .. .. .. .. .. .. .. .. .. .. .. .. .. 624

6. Substitute for Cinchona .. .. .. .. .. .. .. .. .. .. .. .. .. .. [illegible]

